# 380. The first two years of the European VACCELERATE volunteer registry

**DOI:** 10.1093/ofid/ofad500.450

**Published:** 2023-11-27

**Authors:** Jon Salmanton-Garcia, Fiona A Stewart, Sarah Heringer, Janina Leckler, Oliver A Cornely, Zoi Dorothea Pana

**Affiliations:** University Hospital Cologne, Cologne, Germany, Cologne, Nordrhein-Westfalen, Germany; University Hospital Cologne, Cologne, Nordrhein-Westfalen, Germany; University Hospital Cologne, Cologne, Nordrhein-Westfalen, Germany; University Hospital Cologne, Cologne, Nordrhein-Westfalen, Germany; University of Cologne, Cologne, Germany, Cologne, Nordrhein-Westfalen, Germany; European University of Cyprus, Nicosia, Nicosia, Cyprus

## Abstract

**Background:**

The VACCELERATE Volunteer Registry is an active single-entry point for European residents interested in clinical trial participation. It has received funding from the European Union’s Horizon 2020 research and innovation programme (grant agreement 101037867), and the German Federal Ministry of Education and Research (grant BMBF01KX2040). As of today, the VACCELERATE Volunteer Registry is active in 17 countries, and associated to two national registries (France, The Netherlands), with 106,869 registered volunteers overall. Participants provide their contact information, including first and last name and e-mail address, age, gender, pre-existing comorbidities, vaccination status for SARS-CoV-2, COVID-19 history, and maximum distance willing to travel to a clinical trial site, if needed. The registry is open to both adults and children, complying with legal consent requirements. The VACCELERATE Volunteer Registry is active since October 2020.

**Methods:**

Once volunteers sign in on www.vaccelerate.eu/volunteer-registry, data are stored until a request is received from entities managing or

performing clinical trials or epidemiological studies. Based on the inclusion criteria of the respective study, number of volunteers

needed, potential delivery of invitation reminders, and turnaround time is set. Afterwards, matching notifications are delivered.

Eventually, if volunteers autonomously decide to participate in the study, they may contact via email the promoters.

**Results:**

So far, the VACCELERATE Volunteer Registry has been approached to match clinical trials and epidemiological studies with volunteers 28 times. Of those, 17 (61%) studies have been focused on different aspects of SARS-CoV-2, such as finding of the best vaccination scheme, new vaccine testing, or infection point-prevalence. Additionally, new vaccinations against Streptococcus pneumoniae have been the research topic of 6 (21%) studies. Overall, 71,758 study participations have been offered to VACCELERATE volunteers of all ages.

**Conclusion:**

After 2 years since its activation, the VACCELERATE Volunteer Registry has become a reference for study sponsors and clinical trial units to speed up volunteer enrolment in trials. We expect a further increase in volunteer registration, and study requests.

**Disclosures:**

**Oliver A. Cornely, MD PhD**, DZIF: Advisor/Consultant|DZIF: Board Member|DZIF: Grant/Research Support|DZIF: Honoraria|DZIF: Stocks/Bonds

